# Analysis of the (5d^2^+5d6s)–5d6p Transition Arrays of Os VII and Ir VIII, and the 6s ^2^S–6p ^2^P Transitions of Ir IX

**DOI:** 10.6028/jres.100.051

**Published:** 1995

**Authors:** G. J. van het Hof, Y. N. Joshi, J. F. Wyart, J. Sugar

**Affiliations:** Physics Department, St. Francis Xavier University, Box 5000, Antigonish, Nova Scotia, Canada B2G 2W5; Laboratoire Aimé Cotton, CNRS II Bâtiment 505, 91405 Orsay Cedex, France; National Institute of Standards and Technology, Gaithersburg, MD 20899-0001, USA

**Keywords:** energy levels, iridium (Ir VIII), osmium (Os VII), spectra

## Abstract

The spectra of osmium and iridium were photographed in the 300 Å to 1600 Å region on a 3 m normal incidence spectrograph using a triggered spark source. The (5*d*^2^ + 5*d*6*s*)–5*d*6*p* transition arrays of Os VII and Ir VIII were analyzed. All levels of these three configurations in both spectra have been established. There are 77 lines in Os VII and 71 lines in Ir VIII classified. The parametric least squares fitting calculations are used to interpret both spectra. The 6*s* ^2^S_1/2_–6*p* ^2^P_1/2,3/2_ transitions in Ir IX have also been identified.

## 1. Introduction and Experiment

The ground configuration of the seventh spectrum of osmium (Os VII) and the eighth spectrum of iridium (Ir VIII) is 5*d*^2^ and the three lowest excited configurations are the 5*d*6s, 6s^2^ and 5*d*6p. They belong to the Yb I isoelectronic sequence, which has been studied through Re VI [1–5]. The extension of the Yb I sequence is a part of our ongoing project of studying poorly known 5*d*-subshell ionic spectra.

The spectra of osmium and iridium in the 300 Å to 1600 Å wavelength region were excited in a triggered spark discharge and photographed on the 3 m normal incidence spectrograph at St. Francis Xavier University. It is equipped with a holographic grating having a line density of 2400/mm and a plate factor of 1.385 Å/mm in the first order. Osmium or iridium powder was packed into a cavity on the tip of a pure aluminium electrode, which served as the cathode. The anode was a pure aluminium electrode. In later exposures both electrodes contained the sample material. The edges of the electrodes were considerably tapered to avoid low melting point aluminium flowing inwards and blocking the high melting point sample material from getting into the discharge. The electrode gap was set between 2.5 mm to 3 mm. The charging potential was provided by a low inductance 14.3 µF capacitor bank. The discharge conditions were varied primarily by changing the number of turns in a series inductance coil. The charging potential was kept at 4 kV to 5 kV. The lines arising from different ionization stages could thus be reliably discriminated. The exposures above 500 Å were taken with Kodak SWR plates^1^ whereas those below 500 Å were taken on Kodak 101–05 plates. The plates were measured on semiautomatic comparators either at the Zeemen Laboratory in Amsterdam or at the University of New Brunswick (Canada). The internal standards of C, O, Al, and Mg [6] and known osmium and iridium lines [7] were used for plate calibration. The standard uncertainty (i.e., estimated standard deviation) of the wavelength measurements due to the least squares fitted calibration curve is ±0.01 Å. In the region above 1200 Å there were insufficient wavelength standards and the standard uncertainty increased to ±0.02 Å. These uncertainties are due to the standard deviation of the fit of the calibration lines to a polynomial function of position. We estimate the standard uncertainty from systematic effects to be ±0.005 Å. It is kept low by the presence of well-measured internal impurity lines in the same exposure as the desired spectrum. The combined standard uncertainties (i.e., total one standard deviation estimate) in these two regions are thus ±0.011 Å and ±0.021 Å, respectively.

The strongest lines in the Os VII and Ir VIII spectra appeared on the spectrograms as full length images, stretching from anode to cathode. The weaker lines were polar, stretching from the cathode to the middle of the spark gap. We used the length of the lines for roughly estimating their relative intensities.

## 2. Results and Discussion

From fitted calculations of the isoelectronic spectra from Yb I to Re VI for the three configurations 5*d*^2^, 5*d*6*s*, and 5*d*6*p* [1–5], we could accurately predict the scaling factors (the ratio of the least squares fitted (LSF) parameter values to the Hartree-Fock (HF) values of the energy parameters [8].) Nonrelativistic calculations of the transtition arrays with the Cowan code [8] with these parameters showed that for Os VII and Ir VIII the 5*d*^2^–5*d*6*p* array lies in the region 380 Å to 580 Å and 330 Å to 510 Å respectively, and the 5*d*6*s*–5*d*6*p* arrays lay in the regions 850 Å to 1330 Å and 800 Å to 1200 Å respectively. These arrays could be easily identified on the plates because of the good excitation separation. It was also noticed that the 5*d*^2^–5*d*6*p* array was much stronger than the 5*d*6*s*–5*d*6*p* array for both spectra.

With these predictions as a guide we classified 77 lines of Os VII given in Table 1, with only one doubly classified. All lines classified in the Os VII and Ir VIII analyses, except the masked lines or very weak lines, exhibit their respective ionization-stage characteristics. The 71 lines classified in Ir VIII are given in Table 2. The mean deviation of absolute differences of measured wave numbers in cm^−1^ of Os VII lines from those predicted with the final level values is 1.5, and for Ir VIII lines it is 1.6.

All 13 levels of the 5*d*^2^ and 5*d*6*s* even parity configurations and all twelve levels of the 5*d*6*p* odd parity configuration for both Os VII and Ir VIII have been established and are listed in Tables 3, 4 and 5, 6 respectively, along with the two highest eigenvector percentages in *LS* coupling. The standard deviation between calculated and observed levels for the even configurations of Os VII and Ir VIII are 114 cm^−1^ and 124 cm^−1^, and for the odd configuration 354 cm^−1^ and 336 cm^−1^, respectively. One can unambiguously assign *LS* designations to all the levels of even parity for both ions. However, for the odd parity levels none is predominantly ^1^D_2_ or ^3^D_3_. The same was also observed in Re VI [5]. In Tables 4 and 6 two levels are arbitrarily assigned these designations for convenience of classifying the lines (see footnotes b and c in Tables 1 and 2). In the LSF calculations configuration interaction was introduced between the 5*d*^2^, 5*d*6*s* and 6*s*^2^ configurations which improved the fit to some extent. The parameters used in Os VII and Ir VIII are given in Table 7 and Table 8 respectively. We should also point out that the *G*^1^(*dp*) and *G*^3^(*dp*) parameters in the 5*d*6*p* configuration in both spectra were fixed at their HF ratios in the iteration process. When they were not linked the fits improved, giving standard deviations of 222 cm^−1^ and 237 cm^−1^, respectively, but the *G*^3^(*dp*) scaling factors were 0.516 and 0.564, respectively, with almost 12 % uncertainty on their values.

The 5*d*^2^–5*d*5*f* transition array in Os VII and Ir VIII has not been investigated in this research. These transitions lay between 330 Å to 397 Å in Re VI [5] and were much weaker than the (5*d*^2^+5*d*6*s*)–5*d*6*p* transitions. The corresponding transitions in Os VII and Ir VIII are below 300 Å. We intend to undertake this work shortly.

## 3. The 6*s* ^2^*S*_1/2_ Level of Ir IX

**Fig. 1:**
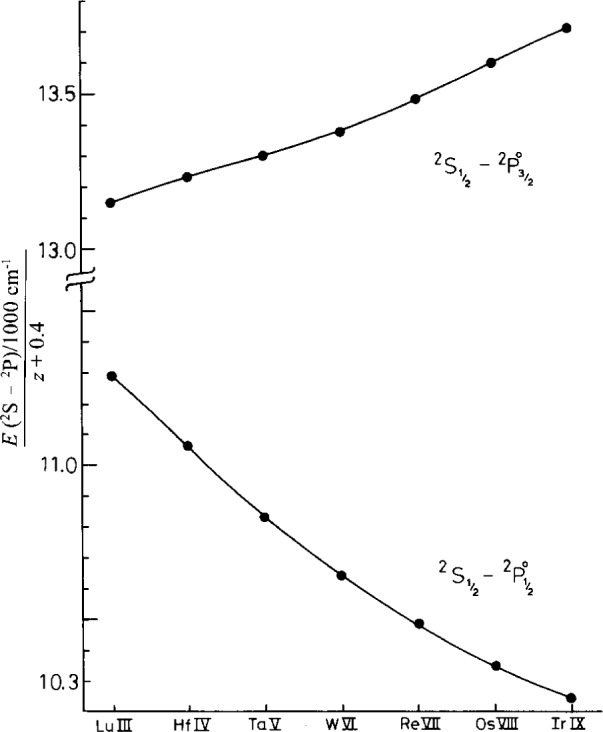
The 6*s* ^2^S–6*p* ^2^P transitions in the Yb II isoelectronic sequence. *Z*_0_ is the spectrum number of the ion. The identified Ir IX lines fit nicely in the isoelectronic sequence extensions.

**Fig. 2:**
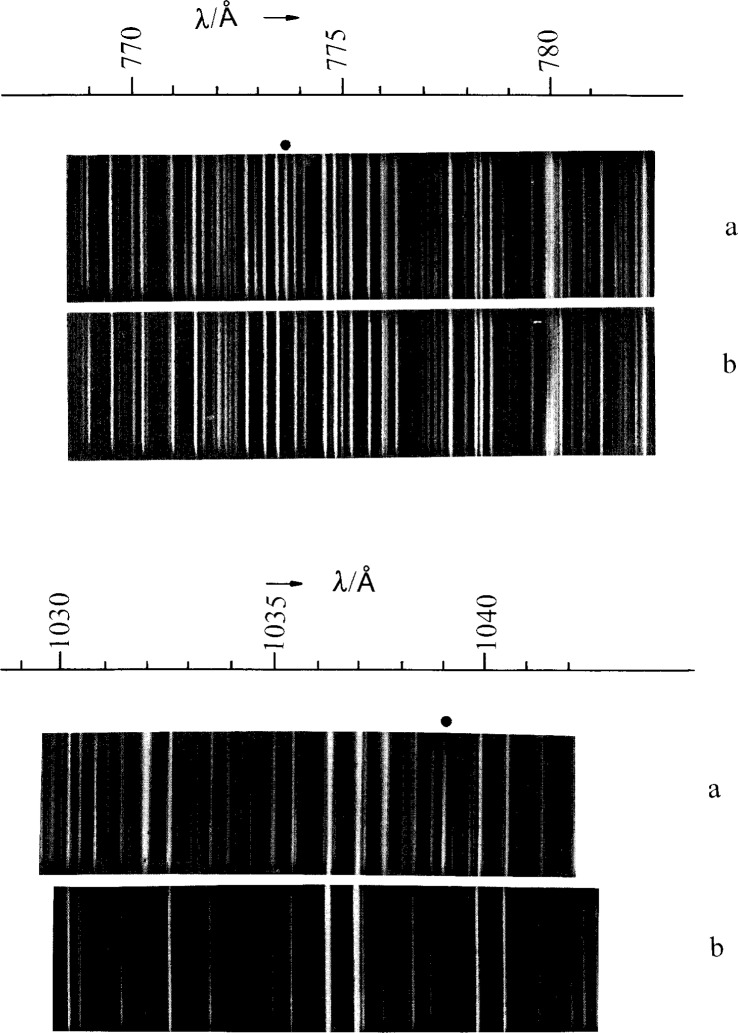
The iridium spectrum in the 770 Å to 780 Å and 1030 Å to 1040 Å regions taken on a 3 m spectrograph using a triggered spark source operated under two experimental conditions: (a) is a high excitation exposure (5 kV, no inductance coil; and (b) is a medium excitation exposure (5 kV, 12 turns of the inductance coil). The Ir IX 6*s* ^2^S–6*p* ^2^P_1/2,3/2_ lines are absent in the medium excitation exposure. They are denoted by ● in the figure.

Kaufman and Sugar [7] identified the 6*s* ^2^S–6*p* ^2^P_3/2,1/2_ transitions in the Yb II sequence from Yb II to Os VIII but did not identify them in Ir IX or in the higher members. Our plates show these lines as well as the six Ir IX lines of 5*d*–6*p* and 5*d*–5*f* identified in Ref. 7. The two 6*s*–6*p* lines of Ir IX were predicted to be at 773.5 Å and 1038 Å by extrapolation of the isoelectronic sequence (see Fig. 1). On examining the plate (see Fig. 2) the lines with correct Ir IX characteristic were identified. They are given in Table 9.

## Figures and Tables

**Table 1:** The (5*d*^2^+5*d*6*s*)–5*d*6*p* transitions of Os VII

*λ*/Å	*σ*/cm^−1^^a^	Intensity	Ch^b^	Even level Odd level	Δλ/Å^c^
391.771	255 251.1	10	7	5*d*^2 3^F_2_–5*d*6*p* ^1^P_1_	−0.008
400.965	249 398.5	10	6	5*d*^2 3^F_2_ –5*d*6p ^1^F_3_	0.005
402.510	248 440.8	50	1	5*d*^2 3^F_2_–5*d*6*p* ^3^P_2_	−0.018
417.700	239 406.5	60	1	5*d*^2 3^P_0_–5*d*6*p* ^1^P_1_	0.004
418.242	239 096.3	65	1	5*d*^2 3^F_3_–5*d*6*p* ^1^F_3_	−0.005
419.945	238 126.2	70	1	5*d*^2 3^F_3_–5*d*6*p* ^3^P_2_	−0.008
420.723	237 686.0	70	8	5*d*^2 3^F_2_–5*d*6*p* ^3^P_1_	−0.006
421.860	237 045.7	60	1	5*d*^2 3^F_3_–5*d*6*p* ^3^F_4_	0.000
422.439	236 720.6	55	1	5*d*^2 3^F_2_–5*d*6*p* ^3^D_3_	0.009
424.073	235 808.5	60	1	5*d*^2 1^D_2_–5*d*6*p* ^1^P_1_	−0.012
428.898	233 155.8	55	1	5*d*^2 3^P_1_–5*d*6*p* ^1^P_1_	−0.015
430.184	232 458.4	58	1	5*d*^2 3^F_2_–5*d*6*p* ^1^D_2_	−0.011
432.237	231 354.7	68	1	5*d*^2 3^F_4_–5*d*6*p* ^1^F_3_	−0.005
434.857	229 960.8	50	1	5*d*^2 1^D_2_–5*d*6*p* ^1^F_3_	−0.007
436.096	229 307.4	70	4	5*d*^2 3^F_4_–5*d*6*p* ^3^F_4_	−0.006
436.708	228 985.8	35	1	5*d*^2 1^D_2_–5*d*6*p* ^3^P_2_	0.000
441.655	226 421.3	70	1	5*d*^2 3^F_3_–5*d*6*p* ^3^D_3_	−0.007
441.826	226 333.4	70	1	5*d*^2 3^P_1_–5*d*6*p* ^3^P_2_	−0.003
444.964	224 737.1	70	1	5*d*^2 3^F_2_–5*d*6*p* ^3^F_3_	0.007
446.849	223 789.3	70	8	5*d*^2 3^F_2_–5*d*6*p* ^3^D_2_	0.002
450.170	222 138.3	75	4	5*d*^2 3^F_3_–5*d*6*p* ^1^D_2_	0.012
450.209	222 118.9	70	5	5*d*^2 3^P_2_–5*d*6*p* ^1^P_1_	0.000
450.758	221 848.6	75	1	5*d*^2 3^P_0_–5*d*6*p* ^3^P_1_	−0.007
457.297	218 676.4	80	4	5*d*^2 3^F_4_–5*d*6*p* ^3^D_3_	0.000
458.208	218 241.5	78	1	5*d*^2 1^D_2_–5*d*6*p* ^3^P_1_	−0.007
458.437	218 132.7	80	4	5*d*^2 1^G_4_–5*d*6*p* ^1^F_3_	−0.012
459.364	217 692.4	70	7	5*d*^2 3^P_1_–5*d*6*p* ^3^P_0_	0.006
460.234	217 281.0	60	1	5*d*^2 1^D_2_–5*d*6*p* ^3^D_3_	0.001
462.380	216 272.1	60	1	5*d*^2 3^P_2_–5*d*6*p* ^1^F_3_	0.005
462.799	216 076.7	60	1	5*d*^2 1^G_4_–5*d*6*p* ^3^F_4_	0.005
463.851	215 586.4	70	1	5*d*^2 3^P_1_–5*d*6*p* ^3^P_1_	−0.005
464.454	215 306.8	70	1	5*d*^2 3^P_2_–5*d*6*p* ^3^P_2_	−0.008
466.358	214 427.4	70	4	5*d*^2 3^F_3_–5*d*6*p* ^3^F_3_	0.012
468.435	213 476.9	70	4	5*d*^2 3^F_3_–5*d*6*p* ^3^D_2_	0.011
469.467	213 007.4	70	1	5*d*^2 1^D_2_–5*d*6*p* ^1^D_2_	0.002
469.797	212 857.7	70	4	5*d*^2 3^F_2_–5*d*6*p* ^3^D_1_	0.008
475.397	210 350.3	40	1	5*d*^2 3^P_1_–5*d*6*p* ^1^D_2_	0.009
479.260	208 654.9	70	1	5*d*^2 3^F_2_–5*d*6*p* ^3^F_2_	0.008
483.819	206 688.7	70	1	5*d*^2 3^F_4_–5*d*6*p* ^3^F_3_	0.006
486.734	205 450.9	70	1	5*d*^2 1^G_4_–5*d*6*p* ^3^D_3_	0.001
487.117	205 289.6	65	7	5*d*^2 1^D_2_ –5*d*6*p* ^3^F_3_	0.017
488.868	204 554.0	30	1	5*d*^2 3^P_2_–5*d*6*p* ^3^P_1_	0.004
489.371	204 343.9	70	1	5*d*^2 1^D_2_ –5*d*6*p* ^3^D_2_	0.005
491.165	203 597.5	50	1	5*d*^2 3^P_2_–5*d*6*p* ^3^D_3_	0.003
495.805	201 692.3	70	1	5*d*^2 3^P_1_–5*d*6*p* ^3^D_2_	−0.001
501.707	199 319.4	50	1	5*d*^2 3^P_2_–5*d*6Ip ^1^D_2_	0.016
506.221	197 542.0	70	8	5*d*^2 1^S_0_–5*d*6*p* ^1^P_1_	−0.017
507.563	197 019.9	70	6	5*d*^2 3^P_0_–5*d*6*p* ^3^D_1_	0.011
516.885	193 466.8	65	1	5*d*^2 1^G_4_ –5*d*6*p* ^3^F_3_	−0.001
521.865	191 620.6	60	1	5*d*^2 3^P_2_–5*d*6*p* ^3^F_3_	−0.018
524.222	190 758.8	10	2	5*d*^2 3^P_1_–5*d*6*p* ^3^D_1_	0.013
524.487	190 662.6	15	2	5*d*^2 3^P_2_–5*d*6*p* ^3^D_2_	0.002
528.505	189 213.0	60	1	5*d*^2 1^D_2_–5*d*6*p* ^3^F_2_	0.004
555.653	179 968.4	75	1	5*d*^2 1^S_0_–5*d*6*p* ^3^P_1_	0.011
569.698	175 531.7	30	1	5*d*^2 3^P_2_–5*d*6*p* ^3^F_2_	0.000
854.600	117 013.8	45	5	5*d*6*s* ^3^D_2_–5*d*6*p* ^3^P_2_	0.000
905.036	110 492.8	50	1	5*d*6*s* ^3^D_1_–5*d*6*p* ^3^P_0_	−0.001
922.672	108 380.9	50	1	5*d*6*s* ^3^D_1_–5*d*6*p* ^3^P_1_	0.005
932.508	107 237.7	30	1	5*d*6*s* ^3^D_3_–5*d*6*p* ^1^F_3_	0.005
932.636	107 223.0	55	1	5*d*6*s* ^1^D_2_–5*d*6*p* ^1^P_1_	0.010
941.023	106 267.3	45	1	5*d*6*s* ^3^D_3_–5*d*6*p* ^3^P_2_	−0.003
				5*d*6*s* ^3^D_2_–5*d*6*p* ^3^P_1_	−0.009
949.584	105 309.3	50	1	5*d*6*s* ^3^D_2_–5*d*6*p* ^3^D_3_	0.003
950.655	105 190.6	70	8	5*d*6*s* ^3^D_3_–5*d*6*p* ^3^F_4_	−0.001
969.440	103 152.3	40	1	5*d*6*s* ^3^D_1_–5*d*6*p* ^1^D_2_	−0.006
986.390	101 379.8	30	1	5*d*6*s* ^1^D_2_–5*d*6*p* ^1^F_3_	−0.001
989.742	101 036.4	50	5	5*d*6*s* ^3^D_2_–5*d*6*p* ^1^D_2_	0.000
995.946	100 407.0	50	8	5*d*6*s* ^1^D_2_–5*d*6*p* ^3^P_2_	0.013
1057.496	945 63.0	20	1	5*d*6*s* ^3^D_3_–5*d*6*p* ^3^D_3_	−0.003
1071.537	933 23.9	40	1	5*d*6*s* ^3^D_2_–5*d*6*p* ^3^F_3_	0.009
1196.743	835 60.1	15	3	5*d*6*s* ^3^D_1_–5*d*6*p* ^3^D_1_	0.007
1210.984	825 77.5	50	1	5*d*6*s* ^3^D_3_–5*d*6*p* ^3^F_3_	0.004
1225.082	816 27.2	50	1	5*d*6*s* ^3^D_3_–5*d*6*p* ^3^D_2_	−0.002
1227.815	814 45.5	30	1	5*d*6*s* ^3^D_2_–5*d*6*p* ^3^D_1_	−0.003
1260.119	793 57.6	50	1	5*d*6*s* ^3^D_1_–5*d*6*p* ^3^F_2_	0.001
1294.622	772 42.6	60	7	5*d*6*s* ^3^D_2_–5*d*6*p* ^3^F_2_	−0.004
1303.446	767 19.7	55	1	5*d*6*s* ^1^D_2_–5*d*6*p* ^3^F_3_	−0.010
1319.811	757 68.4	40	5	5*d*6*s* ^1^D_2_–5*d*6*p* ^3^D_2_	0.001

a Wavenumber of line.

b Ch=character of the line.
1symmetric sharp line.2weak line, less accurate in measurement.3very weak line.4broad line.5suspected line blended with stronger line. (In the table, the wavelength is replaced by value corresponding to the level value difference. The intensity is replaced by an estimated value based on the transition probability).6unresolved from stronger line.7partly blended.8asymmetric line, shaded to shorter wavelength side.9asymmetric line, shaded to longer wavelength side.

c Δλ= *λ* (observed) minus *λ*(derived from levels).

**Table 2:** The (5*d*^2^+5*d*6*s*)–5*d*6*p* transitions in Ir VIII

*λ*/Å	*σ*/cm^−1^^a^	Intensity	Ch^b^	Even level Odd level	Δλ/Å^c^
337.532	296 268.6	15	2	5*d*^2 3^P_0_–5*d*6*p* ^1^P_1_	−0.003
340.105	294 027.0	25	1	5*d*^2 3^F_3_–5*d*6*p* ^1^F_3_	−0.002
341.036	293 224.1	20	8	5*d*^2 3^F_3_–5*d*6*p* ^3^P_2_	−0.002
341.610	292 731.5	30	1	5*d*^2 3^F_2_–5*d*6*p* ^3^P_1_	0.002
342.071	292 336.9	30	1	5*d*^2 3^F_3_–5*d*6*p* ^3^F_4_	−0.004
342.704	291 796.6	30	1	5*d*^2 3^F_2_–5*d*6*p* ^3^D_3_	−0.005
343.538	291 088.9	25	1	5*d*^2 1^D_2_–5*d*6*p* ^1^P_1_	−0.006
346.855	288 305.2	15	2	5*d*^2 3^P_1_–5*d*6*p* ^1^P_1_	−0.007
348.266	287 136.8	15	2	5*d*^2 3^F_2_–5*d*6*p* ^1^D_2_	−0.017
350.768	285 088.6	30	8	5*d*^2 3^F_4_–5*d*6*p* ^1^F_3_	−0.008
351.775	284 273.0	20	2	5*d*^2 1^D_2_–5*d*6*p* ^1^F_3_	−0.015
352.862	283 396.6	75	1	5*d*^2 3^F_4_–5*d*6*p* ^3^F_4_	−0.008
356.281	280 677.0	40	1	5*d*^2 3^P_1_–5*d*6*p* ^3^P_2_	−0.006
358.267	279 121.7	45	1	5*d*^2 3^F_3_–5*d*6*p* ^3^D_3_	−0.002
361.018	276 994.8	30	1	5*d*^2 3^F_2_–5*d*6*p* ^3^F_3_	−0.001
362.492	275 868.4	30	1	5*d*^2 3^F_2_–5*d*6*p* ^3^D_2_	0.005
363.009	275 475.1	40	1	5*d*^2 3^P_0_–5*d*6*p* ^3^P_1_	0.006
364.105	274 646.0	65	1	5*d*^2 3^P_2_–5*d*6*p* ^1^P_1_	−0.005
364.365	274 449.8	80	1	5*d*^2 3^F_3_–5*d*6*p* ^1^D_2_	0.001
369.747	270 455.3	85	4	5*d*^2 1^G_4_–5*d*6*p* ^1^F_3_	−0.014
369.959	270 300.5	60	8	5*d*^2 1^D_2_–5*d*6*p* ^3^P_1_	−0.004
370.130	270 175.7	85	8	5*d*^2 3^F_4_–5*d*6*p* ^3^D_3_	0.002
370.482	269 918.3	60	8	5*d*^2 3^P_1_–5*d*6*p* ^3^P_0_	0.003
371.256	269 355.7	20	1	5*d*^2 1^D_2_–5*d*6*p* ^3^D_3_	0.000
372.087	268 754.5	65	8	5*d*^2 1^G_4_–5*d*6*p* ^3^F_4_	−0.003
373.384	267 820.9	45	8	5*d*^2 3^P_2_–5*d*6*p* ^1^F_3_	−0.002
373.812	267 514.5	65	1	5*d*^2 3^P_1_–5*d*6*p* ^3^P_1_	−0.003
374.497	267 024.5	80	4	5*d*^2 3^P_2_–5*d*6*p* ^3^P_2_	−0.013
377.800	264 690.2	80	1	5*d*^2 1^D_2_–5*d*6*p* ^1^D_2_	−0.006
378.330	264 319.7	85	4	5*d*^2 3^F_3_–5*d*6*p* ^3^F_3_	0.004
379.948	263 194.1	85	4	5*d*^2 3^F_3_–5*d*6*p* ^3^D_2_	0.009
381.001	262 466.8	85	4	5*d*^2 3^F_2_–5*d*6*p* ^3^D_1_	−0.009
381.822	261 902.4	20	2	5*d*^2 3^P_1_–5*d*6*p* ^1^D_2_	−0.002
387.581	258 010.6	90	1	5*d*^2 3^F_2_–5*d*6*p* ^3^F_2_	0.010
391.332	255 537.6	70	8	5*d*^2 1^G_4_–5*d*6*p* ^3^D_3_	0.003
391.584	255 372.8	85	1	5*d*^2 3^F_4_–5*d*6*p* ^3^F_3_	0.010
392.845	254 553.2	65	1	5*d*^2 1^D_2_–5*d*6*p* ^3^F_3_	0.007
393.932	253 850.8	15	7	5*d*^2 3^P_2_–5*d*6*p* ^3^P_1_	0.007
394.582	253 432.8	80	1	5*d*^2 1^D_2_–5*d*6*p* ^3^D_2_	0.005
395.395	252 911.7	40	1	5d^2 3^P_2_–5*d*6*p* ^3^D_3_	0.003
398.967	250 647.3	85	1	5*d*^2 3^P_1_–5*d*6*p* ^3^D_2_	0.006
402.428	248 491.8	70	1	5*d*^2 1^S_0_–5*d*6*p* ^1^P_1_	0.011
402.823	248 248.1	30	7	5*d*^2 3^P_2_–5*d*6*p* ^1^D_2_	−0.006
407.816	245 208.6	80	1	5*d*^2 3^P_0_ –5*d*6*p* ^3^D_1_	−0.003
415.388	240 738.7	75	1	5*d*^2 1^G_4_ –5*d*6*p* ^3^F_3_	0.005
419.965	238 115.3	40	9	5*d*^2 3^P_2_ –5*d*6*p* ^3^F_3_	0.001
421.947	236 996.6	20	7	5*d*^2 3^P_2_ –5*d*6*p* ^3^D_2_	−0.005
424.496	235 573.6	75	7	5*d*^2 1^D_2_ –5*d*6*p* ^3^F_2_	0.013
439.163	227 705.9	20	2	5*d*^2 1^S_0_ –5*d*6*p* ^3^P_1_	0.012
456.339	219 135.4	40	1	5*d*^2 3^P_2_ –5*d*6*p* ^3^F_2_	0.008
506.462	197 448.2	10	2	5*d*^2 1^S_0_ –5*d*6*p* ^3^D_1_	−0.023
743.999	134 408.7	80	7	5*d*6s ^3^D_2_–5*d*6*p* ^3^P_2_	0.002
794.159	125 919.4	70	8	5*d*6*s* ^3^D_1_–5*d*6*p* ^3^P_0_	−0.001
809.641	123 511.5	80	1	5*d*6*s* ^3^D_1_–5*d*6*p* ^3^P_1_	−0.002
813.228	122 966.7	85	8	5*d*6*s* ^1^D_2_–5*d*6*p* ^1^P_1_	0.000
820.016	121 948.8	70	1	5*d*6*s* ^3^D_3_–5*d*6*p* ^1^F_3_	0.006
824.748	121 249.2	70	1	5*d*6*s* ^3^D_2_–5*d*6*p* ^3^P_1_	−0.001
825.452	121 145.8	70	1	5*d*6*s* ^3^D_3_–5*d*6*p* ^3^P_2_	0.002
831.204	120 307.4	85	1	5*d*6*s* ^3^D_2_–5*d*6*p* ^3^D_3_	0.000
831.552	120 257.1	85	1	5*d*6*s* ^3^D_3_–5*d*6*p* ^3^F_4_	0.003
848.179	117 899.6	80	1	5*d*6*s* ^3^D_1_–5*d*6*p* ^1^D_2_	0.002
860.999	116 144.2	80	8	5*d*6*s* ^1^D_2_–5*d*6*p* ^1^F_3_	−0.002
864.771	115 637.6	80	1	5*d*6*s* ^3^D_2_–5*d*6*p* ^1^D_2_	0.001
867.000	115 340.2	75	1	5*d*6*s* ^1^D_2_–5*d*6*p* ^3^P_2_	0.000
1084.055	92 246.2	85	6	5*d*6*s* ^3^D_3_–5*d*6*p* ^3^F_3_	0.004
1097.392	91 125.1	30	1	5*d*6*s* ^3^D_3_–5*d*6*p* ^3^D_2_	−0.007
1099.197	90 975.5	15	2	5*d*6*s* ^3^D_2_–5*d*6*p* ^3^D_1_	0.007
1126.192	88 794.8	30	7	5*d*6*s* ^3^D_1_–5*d*6*p* ^3^F_2_	0.000
1155.627	86 533.1	25	7	5*d*6*s* ^3^D_2_–5*d*6*p* ^3^F_2_	−0.006
1156.853	86 441.4	25	7	5*d*6*s* ^1^D_2_–5*d*6*p* ^3^F_3_	−0.008
1172.084	85 318.1	15	2	5*d*6*s* ^1^D_2_–5*d*6*p* ^3^D_2_	0.009

a,b,c Same as in Table 1.

**Table 3:** Energy levels of the 5*d*^2^, 6*s*^2^ and 5*d*6*s* configurations of Os VII

*J*	*E*(exp)/cm^−1^	*E*(LSF)/cm^−1^^a^	Δ*E*/cm^−1^^b^		Largest *LS* percentages		
0	15 837	15 759	78	91	5*d*^2 3^P	9	5*d*^2 1^S
	57 710	57 686	24	91	5*d*^2 1^S	9	5*d*^2 3^P
		282 345		100	6*s*^2 1^S		
1	22 098	22 067	31	100	5*d*^2 3^P		
	129 301	129 244	57	100	5*d*6*s* ^3^D		
2	0	64	−64	88	5*d*^2 3^F	11	5*d*^2 1^D
	19 444	19 623	−179	47	5*d* ^1^D	44	5*d*^2 3^P
	33 127	33 208	−81	56	5*d*^2 3^P	42	5*d*^2 1^D
	131 416	131 478	−62	82	5*d*6*s* ^3^D	18	5*d*6*s* ^1^D
	148 023	148 009	14	82	5*d*6*s* ^1^D	18	5*d*6*s* ^3^D
3	10 308	10 240	68	100	5*d*^2 3^F		
	142 163	142 172	−9	100	5*d*6*s* ^3^D		
4	10 849	17 931	118	84	5*d*^2 3^F	16	5*d*^2 1^G
	31 274	31 270	4	84	5*d*^2 1^G	16	5*d*^2 3^F

a Energies calculated from LSF parameter values.

b Δ*E* = *E*(exp)–*E*(LSF)

**Table 4:** Energy levels of the 5*d*6*p* configuration of Os VI

*J*	*E*(exp)/cm^−1^	*E*(LSF)/cm^−1^^a^	Δ*E*/cm^−1^^b^		Largest *LS* percentages		
0	239 794	239 438	356	100	^3^P		
1	212 862	212 523	339	69	^3^D	20	^1^P
	237 682	237 621	62	64	^3^P	25	^3^D
	255 246	255 682	−436	69	^1^P	25	^3^P
2	208 659	209 235	−576	69	^3^F	25	^1^D
	223 790	223 670	120	50	^3^D	31	^3^P
	232 453^c^	232 588	−136	39	^3^D	29	^1^D
	248 430	248 204	226	62	^3^P	29	^1^D
3	224 741	224 605	136	58	^3^F	26	^3^D
	236 726^d^	236 783	−57	42	^3^F	31	^3^D
	249 401	249 395	7	57	^1^F	43	^3^D
4	247 354	247 393	−39	100	^3^F		

a Energies claculated from LSF parameter values.

b Δ*E = E*(exp)–*E*(LSF).

c,d These levels are arbitrarily designated in theline-list as ^1^D_2_ and ^3^D_3_, respectively, even though those names have low percentages.

**Table 5:** Energy levels of the 5*d*^2^, 6*s*^2^ and 5*d*6*s* configurations of Ir VIII

*J*	*E*(exp)/cm^−1^	*E*(LSF)/cm^−1^^a^	Δ*E*/cm^−1^^b^		Largest *LS* percentages		
0	17 254	17 170	84	89	5*d*^2 3^P	10	5*d*^2 1^S
	65 022	65 002	19	89	5*d*^2 1^S	10	5*d*^2 3^P
		363 633		100	6*s*^2 1^S		
1	25 221	25 191	30	100	5*d*^2 3^P		
	169 223	169 160	63	100	5*d*6*s* ^3^D		
2	0	80	−80	86	5*d*^2 3^F	12	5*d*^2 3^P
	22 436	22 623	−187	45	5*d*^2 1^D	44	5*d*^2 3^P
	38 878	38 970	−92	54	5*d*^2 3^P	43	5*d*^2 1^D
	171 485	171 551	−67	80	5*d*6*s* ^3^D	20	5*d*6*s* ^1^D
	190 553	190 536	17	80	5*d*6*s* ^1^D	20	5*d*6*s* ^3^D
3	12 672	12 589	83	100	5*d*^2 3^F		
	184 748	184 761	−13	100	5*d*6*s* ^3^D		
4	21 615	21 493	122	79	5*d*^2 3^F	21	5*d*^2 1^G
	36 253	36 233	20	79	5*d*^2 1^G	21	5*d*^2 3^F

a Energies calculated from LSF parameter values.

b Δ*E = E*(exp)–*E*(LSF).

**Table 6:** Energy levels of the 5*d*6*p* configuration of Ir VIII

*J*	*E*(exp)/cm^−1^	*E*(LSF)/cm^−1^^a^	Δ*E*/cm^−1^^b^		Largest *LS* percentages		
0	295 142	294 821	321	100	^3^P		
1	262 461	262 111	350	67	^3^D	21	^1^P
	292 734	292 674	60	63	^3^P	26	^3^D
	313 520	313 901	−380	68	^1^P	26	^3^P
2	258 017	258 595	−578	70	^3^F	25	^1^D
	275 872	275 769	103	49	^3^D	32	^3^P
	287 122	287 204	−82	40	^3^D	29	^1^D
	305 894	305 699	195	60	^3^P	30	^1^D
3	276 994	276 854	140	56	^3^F	26	^3^D
	291 792	291 872	−80	44	^3^F	29	^3^D
	306 697	306 736	−39	54	^1^F	46	^3^D
4	305 005	305 016	−11	100	^3^F		

a Energies calculated from LSF parameter values.

b Δ*E = E*(exp)–*E*(LSF).

c,d These levels are aritrarily designated in the line-listas ^1^D_2_ and ^3^D_3_, respectively, even though those names have low percentages.

**Table 7:** The parameter values of the 5*d*^2^, 6*s*^2^, 5*d*6*s* and 5*d*6*p* configurations of Os VII

Config.	Parameter	LSF/cm^−1^	HF/cm^−1^	LSF/HF
5*d*^2^	*E*_av_(5*d*^2^)	20479 (43)	0	
	*F*^2^(5*d*,5*d*)	62821 (280)	74409	0.844
	*F*^4^(5*d*,5*d*)	43534 (360)	50240	0.867
	*ζ*(5*d*)	4940 (28)	5086	0.971
5*d*6*s*	*E*_av._(5*d*6*d*)	138892 (59)	121995	0.971
	*ζ*(5*d*)	5171 (54)	5300	0.976
	*G*^2^(5*d*,6*s*)	18914 (470)	22876	0.827
6*s*^2^	*E*_av._(6*s*^2^)	261471	261471	1.000^a^
5*d*^2^-5*d*6*s*	*R*^2^(5*d* 5*d*, 5*d* 6*s*)	−23064	−27135	0.850^b^
5*d*^2^-6*s*^2^	*R*^2^(5*d* 5*d*, 6*s* 6*s*)	21175	24912	0.850^b^
	rms deviation	113		
5*d* 6*p*	*E*_av._(5*d*6*p*)	235457 (110)	217170	0.990
	*ζ*(5*d*)	5220 (87)	5351	0.976
	*ζ*(6*p*)	16075 (170)	14388	1.117
	*F*^2^(5*d*,6*p*)	28324(1300)	35970	0.787
	*G*^1^(5*d*,6*p*)	9628 (560)	13430	0.717
	*G*^3^(5*d*,6*p*)	8825 (510)	12310	0.717^c^
	rms deviation	353		

a *E*_av._(6*s*^2^) is kept at HF value relative to the ground state.

b Interaction parameters are fixed at 85% of HF values.

c *G*^k^(5*d*,6*p*) are fixed at HF ratio.

**Table 8:** The parameter values of the 5*d*^2^, 6*s*^2^, 5*d* 6*s* and 5*d*6*p* configurations of Ir VIII

Config.	Parameteter	LSF/cm^−1^	HFcm^−1^	LSF/HF
5*d*^2^	*E*_av._(5*d*^2^)	23 911 (46)	0	
	*F*^2^(5*d*,5*d*)	67 380 (310)	79314	0.850
	*F*^4^(5*d*,5*d*)	47 186 (410)	53773	0.878
	*ζ* 5*d*	5 990 (30)	6116	0.979
5*d*6*s*	*E*_av_.(5*d* 6*s*)	180 465 (64)	160568	0.975
	*ζ* (5*d*)	6 240 (58)	6342	0.984
	*G*^2^(5*d*,6*s*)	19 450 (520)	23323	0.834
6*s*^2^	*E*_av_(6*s*^2^)	363 329	339418	1.000^a^
5*d*^2^–5*d*6*s*	*R*^2^(5*d* 5*d*, 5*d* 6*s*)	−23 121	−27201	0.850^b^
5*d*^2^–6*s*^2^	*R*^2^(5*d* 5*d*, 6*s* 6*s*)	21 454	25240	0.850^b^
	rms deviation	122		
5*d*6*p*	*E*_0_(5*d*6*p*)	290 176 (100)	269 076	0.990
	*ζ* (5*d*)	6 291 (82)	6 394	0.984
	*ζ* (6*p*)	19 709 (160)	17 929	1.099
	*F*^2^(5*d*,6*p*)	31 325 (1220)	38 920	0.805
	*G*^1^(5*d*,6*p*)	10 161 (540)	13 860	0.733
	*G*^3^(5*d*,6*p*)	9 516 (500)	12 980	0.733^c^
	rms deviation	336		

a *E*_av(6_*_s_*^2^_)_ is kept at HF value relative to the ground state.

b Interaction parameters are fixed at 85 % of HF values.

c *G^k^*_(5_*_d_*_,6_*_p_*_)_ are fixed at HF ratio.

**Table 9:** The 6*s* ^2^S–6*p* ^2^P transitions in Ir IX

*λ* /Å	*I*	*σ* /cm^−1^	Classification^a^
773.616	80	129263	6*s* ^2^S_1/2_–6*p* ^2^P_3/2_
1039.041	70	96242	6*s* ^2^S_1/2_–6*p* ^2^P_1/2_

a This places the 6*s* S_1/2_ term at 195 096 cm^−1^.

## Footnotes

1 Certain commercial equipment, instruments, or materials are identified in this paper to foster understanding. Such identification does not imply recommendation or endorsement by the National Institute of Standards and Technology, nor does it imply that the materials or equipment identified are necessarily the best available for the purpose.
